# The Association between Point-of-Sale Advertising Bans and Youth Experimental Smoking: Findings from the Global Youth Tobacco Survey (GYTS)

**DOI:** 10.3934/publichealth.2015.4.832

**Published:** 2015-12-18

**Authors:** Ce Shang, Jidong Huang, Qing Li, Frank J. Chaloupka

**Affiliations:** 1 Health Policy Center, Institute for Health Research and Policy, University of Illinois at Chicago, Chicago, IL 60608, USA; 2 Department of Economics, University of Illinois at Chicago, Chicago, IL 60608, USA

**Keywords:** Point-of-sale advertising ban, experimental smoking, youth, Global Youth Tobacco Survey, MPOWER

## Abstract

Background and Objectives: while existing research has demonstrated a positive association between exposure to point-of-sale (POS) tobacco advertising and youth smoking, there is limited evidence on the relationship between POS advertising restrictions and experimental smoking among youth. This study aims to fill this research gap by analyzing the association between POS advertising bans and youths' experimental smoking. Methods: Global Youth Tobacco Surveys from 130 countries during 2007–2011 were linked to the WHO “MPOWER” tobacco control policy measures to analyze the association between POS advertising bans (a dichotomous measure of the existence of such bans) and experimental smoking using weighted logistic regressions. All analyses were clustered at the country level and controlled for age, parents' smoking status, GDP per capita, and country-level tobacco control scores in monitoring tobacco use, protecting people from smoke, offering help to quit, warning about the dangers of tobacco, enforcing promotion/advertising bans, and raising taxes on tobacco. Results: The results suggest that a POS advertising ban is significantly associated with reduced experimental smoking among youth (OR = 0.63, *p* < 0.01), and that this association is seen for both genders (boys OR = 0.74, *p* < 0.1; girls OR = 0.52, *p* < 0.001). Conclusions: POS advertising bans are significantly associated with reduced experimental smoking among youth. Adopting POS advertising bans has the potential to reduce tobacco use among their youth in countries currently without such bans.

## 1. Introduction

As the regulation of tobacco advertising in traditional media such as television and print has become more restrictive in the US, point-of-sale (POS) advertising is increasingly used by the tobacco industry to market their products to consumers, particularly to youth and young adults [1]–[7]. Studies have shown that more POS tobacco promotion, such as advertising and preferred ways of display, is observed in stores located near schools or in communities with a higher proportion of potential youth consumers [8]–[10]. Recent systematic reviews further conclude that exposure to POS tobacco advertising is linked to increased susceptibility to smoking, experimental smoking, and smoking participation among children and young people, raising concerns about its adverse public health consequences [11],[12].

The evidence from existing literature underscores the need of regulating POS promotion. Studies have shown that POS promotion induces youth and young adults to experiment with smoking, which could lead to a lifelong addiction [8]–[9],[11]–[34]. Specifically, a number of studies have shown that youth and young adults who are exposed to POS promotion are 1.1–2.7 times more likely to experiment with smoking [11],[12],[33],[34]. Legislations banning POS promotion, including advertising and displays, may have significant public health benefits by effectively reducing smoking among young people.

However, despite the importance of regulating POS promotion, such policies are lacking in many countries. According to the World Health Organization (WHO), as of 2012, only 67 out of 225 countries have banned POS advertising, with just around 25% of the world population protected by such bans [35]. There are even fewer countries banning POS displays, with 17 countries having such bans primarily from the developed world such as Canada and Australia. In the US, the 2009 Family Smoking Prevention and Tobacco Control Act (FSPTCA) gave the US Food and Drug Administration (FDA) authority to regulate the manufacture, distribution and marketing of tobacco products, including POS promotion. However, to date, other than bans on free cigarette samples and selling tobacco products via vending machines, there are almost no other regulations of POS market and promotion in place[36].

In this context, empirical evidence that examines the effectiveness of POS promotion bans in reducing smoking is highly warranted. Whereas there are several studies that investigated the effects of POS promotion or display bans, the findings are inconsistent [11],[37]–[40]. Specifically, two studies used Irish data to examine the effects of banning POS displays and advertising and did not find any significant changes in smoking behavior or cigarette sales after the implementation of the ban [38],[39]. Another study employed longitudinal data from the US, UK, Australia, and Canada to study the effects of POS display bans, and found such bans lowering impulse purchases and non-usual brand purchase [37].

Given the very limited and inconsistent empirical evidence, more research is needed to examine the effectiveness of regulating POS promotion in reducing smoking. This study contributes to the existing literature by utilizing data from 130 countries (see the Appendix) to examine the association between POS advertising bans and youth experimental smoking. The study was conducted using cross-country comparison and adjusting for individual-level demographic characteristics and country-level GDP per capita and tobacco control environment.

## 2. Materials and Method

### 2.1. Data and measures

#### 2.1.1. MPOWER

WHO monitors the implementation of six proven tobacco control measures (MPOWER) in its 196 participating countries: M (Monitor tobacco use), P (protect people from smoke), O (offer help to quit), W (warn about the dangers of tobacco), E (enforce bans on tobacco marketing), and R (raise taxes on tobacco). For each of the above six policy dimensions, the MPOWER data provides a composite score that measures its overall strength (a score of 1 represents a lack of data and a score 2–5 represents weakest to strongest policy strength) and the detailed information of individual policies in these policy dimensions. This data in years 2007–2008, 2010, and 2012 was available to us. In this analysis, we utilized the MPOWER information about advertising bans at the point of sale to examine their association with experimental smoking among youth, after controlling for the overall tobacco control environment using the six composite scores.

Specifically, the existence of a POS advertising ban is measured using a dichotomous variable with countries that have such a ban coded with 1 and those that do not coded with 0. For countries that had POS advertising bans in our study period (2007–2011), we verified the date when the POS advertising ban was implemented, and constructed yearly time series data of these bans between 2007 and 2012 that reflects the policy in place. Because the six composite scores in years 2009 and 2011 are not available from the MPOWER data, the scores in the prior years (2008 and 2010) were used for those two missing data points in the analyses, by assuming that there was no policy change over those two years. Furthermore, given that a composite score of 1 represents a lack of data rather than the strength of the policy, to separate its effects from the effects of policy strength, we controlled for a dummy variable that indicates the lack of data in addition to the six composite scores.

#### 2.1.2. Global youth tobacco survey (GYTS)

GYTS is a school-based survey designed to monitor tobacco use among youth on a global basis and to guide the implementation and evaluation of tobacco control policies. Among the countries surveyed during 2007–2011, 130 had MPOWER policy information and were used in this analysis. In addition, a majority of 107 countries were surveyed only once and 17 countries were surveyed two to three times, with the rest 6 countries surveyed more than once but in different subnational regions. As a result, year and country fixed effects were not controlled for because the data lacks policy variation over time within the same country. Following previous studies [11],[31],[33],[34], experimental smoking was identified using the question “Have you ever tried a cigarette, even one or two puffs?” Those who had never tried a cigarette were coded with 0 and those who had were coded with 1. In addition, the GYTS contains information about respondents' age (≤ 11-referent, 12,13,14,15,16, ≥ 17, and missing), gender, and their parents' smoking status (neither-referent, both, father only, mother only, and missing/don't know), which were controlled for in the analyses. In Table 1, we show the definitions of country-level GDP per capita from the World Development Indicators (WDI) data and policy variables from the MPOWER data and individual-level experimental smoking status and demographic variables from the GYTS. Finally, using country and year identifiers, GDP per capita and MPOWER policy variables were linked to the GYTS data to carry out the analyses.

**Table 1.** Variable Definition for Country—level Policies and Individual-level Experimental Smoking and Demographic Characteristics.

Variable Name	Description
*Country-level policy variables-MPOWER*	
POS ad ban	Binary variable for banning advertising at the point of sale.
M score	Categorical variable; = 1 if no known data, or no recent data (since 2007) or data that is not both recent and representative (national population); = 2 if there are recent and representative data for either adults or youth; = 3 if there are recent and representative data for both adults and youth; = 4 if there are recent, representative and periodic data (at least every 5 years) for both adults and youth.
P score	Categorical variable; = 1 if data not reported or not categorized; = 2 if up to two public places completely smoke-free; = 3 if three to five public places completely smoke-free; = 4 if six to seven public places completely smoke-free; = 5 if all public places completely smoke-free (or at least 90% of the population covered by complete subnational smoke-free legislation; excluding pubs and bars where these are illegal)
O score	Categorical variable; = 1 if data not reported; = 2 if none; = 3 if there are nicotine replacement therapy (NRT) and/or some cessation services (neither cost-covered); = 4 of there are NRT and/or some cessation services (at least one of which is cost-covered); = 5 if there are national quit lines, and both NRT and some cessation services cost-covered
W score	Categorical variable; = 1 if data not reported; = 2 if no warnings or small warnings (< 30%); = 3 if medium size warnings (30%–49%) missing one or more appropriate characteristics or large warnings (≥ 50%) missing four or more appropriate characteristics; = 4 if medium size warnings (30%–49%) with all seven appropriate characteristics or large warnings (≥ 50%) missing one or more appropriate characteristics; = 5 if large warnings (≥ 50%) with all seven appropriate characteristics
E score	Categorical variable; = 1 if data not reported; = 2 if none; = 3 if complete absence of ban, or ban that does not cover national TV, radio or print media; = 2 if ban on national TV, radio and print media only; = 3 if ban on national TV, radio and print media as well as on some but not all other forms of direct and/or indirect advertising; = 4 if ban on all forms of direct and indirect advertising.
R score	Categorical variable; = 1 if data not reported; = 2 if ≤ 25% of retail price is tax; = 3 if 26–50% of retail price is tax; = 4 if 51–75% of retail price is tax; = 5 if >75% of retail price is tax.
GDP per capita	Inflation-adjusted real GDP per capita in thousand dollars, base year 2005.
*Individual-level variables*	
Experimental Smoking	Indicator equals 1 if the respondent ever smoked cigarettes, 0 otherwise
Age dummies	Binary indicators for 8 age categories (≤ 11-referent) from age 12 to 16, and ≥ 17.
Parents' smoking status	Binary indicator for 5 categories of parents' smoking status (neither as referent), both, father only, mother only, and missing/don't know.

### 2.2. Methodology

Logistic regressions were employed to analyze the association between POS advertising bans and experimental smoking. Following other cross-country studies [43]–[45], GYTS final weights rescaled to the sample size of each country were used as analytical weights throughout the analyses. Because numerous studies have documented the significant difference in smoking by gender, in addition to a pooled analyses of both girls and boys, the analyses were also conducted by stratifying the sample by gender to examine gender differences in policy impacts. The analytical model can be presented using the following equation:

$$Experiment_{it}=POS_{it}+MPOWER_{it}+X_{it}+e_{it}$$ (1)

Where POS_it_ is the dichotomous variable indicating the existence of a POS advertising ban. MPOWER_it_ is a vector of country-level variables, which are the six composite policy scores and GDP per capita. X_it_ is a vector of individual-level sociodemographic variables, which are age dummies, gender (only in pooled analyses), and parents' smoking status, with details presented in the Data and Measures Section. As noted above, year and country fixed effects were not included due to the lack of the time dimension in the data. Because the MPOWER scores in the prior years were used for missing data points in years 2009 and 2011, by assuming that there was no policy change over those two years. We conducted sensitivity analyses by using the scores from the subsequent years (2010 and 2012), and by randomly assigning the scores from either the prior or the subsequent year, to examine whether results change significantly by these alternative ways of reconstructing tobacco control environment measures. We also conducted another set of sensitivity analyses by dropping the 23 countries that had been surveyed more than once during 2007–2011 to examine whether results are sensitive to sample compositions. All analyses were conducted using Stata v. 13.0.1. Robust standard errors clustering at the country level were obtained.

## 3. Results

Table 2 shows summary statistics for the analytical samples (all, boys, and girls) weighted using GYTS final weights rescaled to the sample size of each country. After dropping respondents whose gender or status of experimental smoking is missing, the sample sizes of both genders, boys, and girls are 571,505; 277,945; and 293,560 respectively. The prevalence of experimental smoking was 31% among youth, 37% among boys, and 26% among girls. 24% of respondents lived in a country with POS advertising bans in place. The age distribution shows that each age category accounts for about 10–20% of the sample. Parents' smoking status suggests that, while about 60% of respondents reported that neither parents smoke, 7% reported that both parents smoke, 25% reported that their father smokes, and 4% reported that their mother smokes. The means of MPOWER scores are mostly between 2 and 3, suggesting that on average there are tobacco control policies in place, with some countries implementing none and some countries implementing policies with a higher strength.

**Table 2.** Weighted Summary Statistics.

		All		Boys		Girls	
		Mean	[95% C.I.]	Mean	[95% C.I.]	Mean	[95% C.I.]
Experimental Smoking		0.314	[0.275, 0.353]	0.371	[0.334, 0.408]	0.258	[0.212, 0.304]
POS ad ban		0.239	[0.144, 0.334]	0.234	[0.142, 0.327]	0.244	[0.147, 0.340]
Age dummies							
	≤ 11	0.060	[0.037, 0.084]	0.069	[0.044, 0.094]	0.052	[0.028, 0.075]
	12	0.089	[0.073, 0.104]	0.082	[0.067, 0.096]	0.096	[0.077, 0.114]
	13	0.187	[0.164, 0.210]	0.176	[0.154, 0.199]	0.198	[0.173, 0.222]
	14	0.227	[0.207, 0.246]	0.217	[0.198, 0.237]	0.236	[0.215, 0.256]
	15	0.204	[0.186, 0.223]	0.203	[0.185, 0.221]	0.206	[0.186, 0.226]
	16	0.121	[0.104, 0.138]	0.128	[0.111, 0.145]	0.114	[0.096, 0.131]
	≥ 17	0.069	[0.046, 0.092]	0.082	[0.057, 0.107]	0.057	[0.036, 0.077]
	missing	0.043	[−0.016, 0.101]	0.042	[−0.013, 0.098]	0.043	[−0.018, 0.105]
Parent smoking status							
neither		0.566	[0.530, 0.601]	0.569	[0.535, 0.603]	0.563	[0.526, 0.599]
both		0.070	[0.056, 0.084]	0.069	[0.055, 0.082]	0.071	[0.057, 0.085]
father only		0.253	[0.220, 0.286]	0.251	[0.220, 0.282]	0.255	[0.221, 0.290]
mother only		0.040	[0.030, 0.050]	0.038	[0.029, 0.048]	0.042	[0.032, 0.053]
don't know		0.071	[0.039, 0.103]	0.073	[0.042, 0.104]	0.069	[0.036, 0.102]
M score		2.831	[2.653, 3.009]	2.830	[2.650, 3.010]	2.832	[2.655, 3.009]
M score missing		0.089	[0.037, 0.141]	0.092	[0.039, 0.144]	0.087	[0.036, 0.138]
P score		2.529	[2.317, 2.740]	2.526	[2.319, 2.734]	2.531	[2.315, 2.747]
P score missing		0.069	[−0.014, 0.151]	0.069	[−0.013, 0.151]	0.069	[−0.015, 0.152]
O score		3.308	[3.174, 3.443]	3.307	[3.174, 3.440]	3.310	[3.173, 3.446]
W score		2.680	[2.427, 2.933]	2.673	[2.423, 2.922]	2.688	[2.431, 2.945]
E score		3.136	[2.898, 3.374]	3.138	[2.901, 3.375]	3.133	[2.894, 3.373]
R score		3.385	[3.197, 3.573]	3.379	[3.191, 3.567]	3.391	[3.202, 3.580]
R score missing		0.017	[−0.005, 0.038]	0.017	[−0.005, 0.039]	0.016	[−0.005, 0.038]
GDP per capita		4.750	[3.655, 5.846]	4.701	[3.603, 5.798]	4.800	[3.700, 5.899]
N		571,505		277,945		293,560	

Table 3 contains the estimates of the association between POS advertising bans and experimental smoking after adjusting for age, parents' smoking status, and country-level GDP per capita and policy environment. Results suggest that the existence of POS advertising bans is significantly associated with reduced experimental smoking among youth (OR = 0.63, *p* < 0.01), and that this association is seen for both genders (boys OR = 0.74, *p* < 0.1; girls OR = 0.52, *p* < 0.001). Compared with youth aged 11 or younger, those aged 15 or older are more likely to have experimented with smoking (*p* < 0.001). In addition, youth who have at least one smoking parent are more likely to have experimented with smoking (*p* < 0.001), compared with those whose parents do not smoke.

**Table 3.** Weighted Logistic Regressions, the Association between POS Ad Bans and Experimental Smoking.

	All		Boys		Girls	
	AOR	[95% C.I.]	AOR	[95% C.I.]	AOR	[95% C. I.]
**POS ad ban**	0.632**	[0.454,0.881]	0.744+	[0.532,1.041]	0.516***	[0.353,0.753]
Age dummies						
≤ 11	REF		REF		REF	
12	0.746*	[0.575,0.968]	0.834	[0.661,1.053]	0.610**	[0.437,0.852]
13	0.722***	[0.595,0.877]	0.802**	[0.664,0.969]	0.594***	[0.467,0.755]
14	0.988	[0.809,1.206]	1.105	[0.908,1.345]	0.809+	[0.635,1.031]
15	1.390**	[1.132,1.706]	1.556***	[1.291,1.876]	1.141	[0.881,1.478]
16	1.753***	[1.391,2.209]	1.922***	[1.563,2.365]	1.479*	[1.091,2.004]
17	2.010***	[1.527,2.646]	2.263***	[1.791,2.859]	1.600*	[1.062,2.412]
missing	0.714	[0.403,1.267]	0.854	[0.471,1.546]	0.502*	[0.272,0.927]
Parents' smoking						
neither	REF		REF		REF	
both	3.140***	[2.734,3.606]	2.618***	[2.262,3.031]	3.727***	[3.161,4.394]
father only	1.625***	[1.495,1.767]	1.715***	[1.574,1.868]	1.527***	[1.322,1.763]
mother only	2.971***	[2.564,3.444]	2.459***	[2.129,2.841]	3.494***	[2.901,4.208]
don't know	1.381*	[1.008,1.892]	1.272+	[0.980,1.650]	1.545*	[1.038,2.299]
M score	0.938	[0.759,1.159]	0.883	[0.708,1.102]	1.003	[0.803,1.253]
M score missing	0.525**	[0.336,0.821]	0.565*	[0.359,0.888]	0.430**	[0.237,0.780]
P score	1.137	[0.943,1.372]	1.119	[0.931,1.345]	1.16	[0.928,1.452]
P score missing	1.16	[0.550,2.446]	0.988	[0.481,2.027]	1.388	[0.610,3.159]
O score	1.031	[0.835,1.271]	1.029	[0.841,1.260]	1.029	[0.797,1.328]
W score	1.076	[0.925,1.251]	1.052	[0.910,1.215]	1.093	[0.910,1.313]
E score	1.034	[0.874,1.223]	1.049	[0.902,1.221]	1.011	[0.807,1.267]
R score	1.470***	[1.264,1.710]	1.430***	[1.231,1.662]	1.533***	[1.260,1.865]
R score missing	2.788**	[1.327,5.857]	2.746*	[1.151,6.554]	2.732*	[1.183,6.305]
GDP per Capita	1.038***	[1.016,1.060]	1.034**	[1.013,1.055]	1.043***	[1.020,1.067]

+ < 0.1, *< 0.05 , **< 0.01, *** < 0.001 in two-tailed test

In summary, these results suggest that POS advertising bans are associated with less experimental smoking among youth, which are in line with the existing evidence that POS advertising attracts young people to experiment with smoking and establish addiction [8]–[9],[11]–[34]. In Table 4 Column 1, we further present the association between POS advertising bans and experimental smoking in terms of marginal effects and elasticity. These results show that the implementation of POS advertising bans is associated with an 8 percentage-point or a 31% reduction (*p* <0.01) in experimental smoking among youth. In Table 4, we further reported sensitivity analyses by using the MPOWER scores from the subsequent year (Column 2), and by randomly assigning the scores from either the prior or the subsequent year (Column3). These results of sensitivity analyses are very close to those in Column 1. Moreover, we conducted another set of sensitivity analyses by dropping the 23 countries where the GYTS was conducted more than once during 2007–2011 and the results also remain very similar (not shown).

**Table 4.** Marginal Effects and Elasticity, the Association between POS Ad Bans and Experimental Smoking, Both Sexes, Sensitivity Analysis included.

	Prior	Subsequent	Random Assignment
Marginal Effect	−0.087**	−0.094**	−0.090**
(S.E.)	(0.032)	(0.031)	(0.031)
Elasticity	−0.314**	−0.342**	−0.326**
(S.E.)	(0.115)	(0.112)	(0.111)

+ < 0.1, *< 0.05, **< 0.01, ***< 0.001 in two-tailed test. When conducting analyses reported in Table 3, we used prior information to fill in missing policy scores in years 2009 and 2011 (Column1).

## 4. Discussion

Existing literature has documented that POS advertising increases youth smoking [8]–[9], [11]–[34]. However, little is known about the effects of POS promotion bans on youth smoking. This study contributes to the existing literature by analyzing the association between POS advertising bans and experimental smoking among youth using GYTS data from 130 countries. The results indicate that POS advertising bans are significantly associated with less experimental smoking among youth (OR = 0.63, *p* < 0.01), and that this association is seen for both genders. The analyses also show that boys, youth aged 15 or older, and those who have at least one parent smoking, are more likely to have experimented with smoking.

Our findings are consistent with existing studies that link POS advertising exposure to experimental smoking. [8]–[9],[11]–[34] Taken together, these studies clearly illustrate that tobacco POS advertising entices youth to experiment with smoking, and policies prohibit such advertising may significantly reduce the likelihood of experimental smoking among youth. These findings also corroborate an existing study that analyzed the effects of POS display bans on adult tobacco purchase behavior [37]. Together, these studies suggest that a comprehensive POS marketing ban that prohibits POS advertising and display may significantly reduce smoking in the general population.

As the FDA is moving toward finalizing its deeming rule of tobacco products, our findings provide important and timely evidence to inform about FDA's future regulatory actions on POS marketing. Given that POS advertising is more prevalent in locations where youth live and the potential effectiveness of POS promotion bans in reducing youth smoking [8]–[10], policies that prohibit POS advertising and display could have significant public health benefits and serve as an effective tobacco control means to achieve the goals of Healthy People 2010 to reduce youth smoking to 5.8% [41]. Given that our analyses were conducted using international data from 130 countries, our findings also provide evidence to support the full implementation of WHO Framework Convention on Tobacco Control (FCTC) guidelines regarding tobacco advertising, promotion, and sponsorship [42].

This study has limitations. First, variations in POS advertising bans in our study came from differences across countries, and we were unable to take advantage of the variations in POS advertising bans over time within the same country. This is because a large proportion of the GYTS countries do not have multiple waves of survey in our study period. Second, some demographic variables such as allowances and peer influences are not consistently measured across countries in the GYTS data and thus were not controlled for in the analyses. GYTS is a school-based survey that does not capture tobacco use behavior by youth who are not at school. In addition, some GYTS surveys are not nationally representative. Therefore, we conducted both un-weighted analyses and weighted analyses using rescaled weights, which produced very similar results. We reported results from weighted analyses because they are widely used when multiple surveys with different sampling methods and survey designs are pooled together to carry out the analyses [43]–[45]. Last, the experimental smoking measure was constructed using self-reported information and may contain measurement errors.

Despite these limitations, our study demonstrated the association between tobacco POS advertising bans and a reduction in youth experimental smoking. Future research can build on our study and further examine the effects of such bans on smoking participation, cigarette consumption, and quitting among the general population.

## 5. Conclusion

Results from this study demonstrate that tobacco POS advertising bans are associated with lower probability of experimental smoking among youth. Our findings can inform the FDA about their potential future regulations on tobacco POS advertising. This study also provides evidence to inform policy makers worldwide about the potential effectiveness of tobacco POS advertising bans in reducing youth smoking and to support the implementation of the guidelines of WHO FCTC regarding tobacco POS advertising, promotion, and sponsorship.

## Supplementary (if necessary)

Appendix:

GYTS analysis (130 countries)

Albania, Algeria, Antigua & Barbuda, Argentina, Armenia, Azerbaijan

Bangladesh, Barbados, Belize, Bhutan, Bosnia and Herzegovina, Botswana, Brazil, Bulgaria, Burkina Faso, Burundi

Cambodia, Cameroon, Cape Verde, Central African Republic, Chile, Colombia, Comoros, Congo, Costa Rica, Côte d'Ivoire, Croatia, Cuba, Czech Republic.

Democratic Republic of the Congo, Djibouti, Dominica

Ecuador, Egypt, El Salvador, Equatorial Guinea, Estonia

Fiji

Gambia, Georgia, Ghana, Grenada, Guatemala, Guinea, Guinea Bissau, Guyana

Hungary

India, Indonesia, Iran, Iraq, Italy

Jordan

Kazakhstan, Kenya, Kiribati, Kuwait, Kyrgyzstan

Laos, Latvia, Lebanon, Lesotho, Liberia, Libya, Lithuania

Macedonia, Madagascar, Malawi, Malaysia, Maldives, Mali, Mauritania, Mauritius, Mexico, Micronesia, Moldova, Morocco, Mozambique

Namibia, Nepal, New Zealand, Niger, Nigeria

Oman

Pakistan, Panama, Papua New Guinea, Paraguay, Peru, Philippines, Poland

Qatar

Romania, Rwanda

Saint Kitts and Nevis, Saint Lucia, Saint Vincent, Samoa, Sao Tome and Principe, Saudi Arabia, Senegal, Serbia, Seychelles, Sierra Leone, Slovakia, Slovenia, Solomon Islands, South Africa, South Korea, Sri Lanka, Sudan, Suriname, Swaziland, Syria

Tanzania, Thailand, Togo, Tonga, Trinidad and Tobago, Tunisia

Uganda, Ukraine, Uruguay, Uzbekistan

Vanuatu, Venezuela, Vietnam

Yemen

Zambia, Zimbabwe
